# Molecular Signatures of Aeroallergen Sensitization in Pediatric Populations: A Comparative Study Across Spanish Cities

**DOI:** 10.3390/ijms26072963

**Published:** 2025-03-25

**Authors:** Ana Martínez-Cañavate, María Mesa-Del-Castillo, Francisco Carballada, Cristina Rivas-Juesas, José Ángel Porto, Cristina Blasco, Montserrat Álvaro-Lozano, Jaime Lozano, Julián Andrés Manrique, María José Martínez, Tania Galán, Gema Domingo, Laura Marín, Pilar Vega, Raquel López-Rodríguez, Práxedes Solano Galán, Yolanda Aliaga, Fernando Pineda, Miguel Tortajada-Girbés

**Affiliations:** 1 Hospital Universitario Virgen de las Nieves, 18014 Granada, Spain; anamartinezcanavate@gmail.com (A.M.-C.); lamarinlo@gmail.com (L.M.); 2 Hospital Universitario de Móstoles, 28935 Madrid, Spain; mesadelcastillomaria@hotmail.com (M.M.-D.-C.); pilarvega20@gmail.com (P.V.); 3 Hospital Universitario Lucus Augusti, 27003 Lugo, Spain; francisco.carballada.gonzalez@sergas.es (F.C.); raquel.lopez.rodriguez2@sergas.es (R.L.-R.); 4 Hospital de Sagunto, Port de Sagunt, 46520 Valencia, Spain; crisrijue@gmail.com (C.R.-J.); praxedesolano1985@gmail.com (P.S.G.); 5 Hospital HM Policlínico La Rosaleda, 15701 Santiago de Compostela, Spain; jportoa@hotmail.com; 6 Hospital Miguel Servet, 50009 Zaragoza, Spain; cris.blasco@hotmail.com (C.B.); yolaaliaga@hotmail.com (Y.A.); 7 Hospital Sant Joan de Déu, 08950 Barcelona, Spain; montserrat.alvaro@sjd.es (M.Á.-L.); jaime.lozano@sjd.es (J.L.); jandres.manrique@sjd.es (J.A.M.); 8 Applied Science, Inmunotek S.L., Alcalá de Henares, 28805 Madrid, Spain; mjmartinez@inmunotek.com (M.J.M.); tgalan@inmunotek.com (T.G.); 9 Hospital Universitario y Politécnico La Fe, 46026 Valencia, Spain; dagema.96@gmail.com (G.D.); tortajadamig@gmail.com (M.T.-G.)

**Keywords:** aerobiology, exposome, allergens, allergic rhinitis, allergic asthma, climate change

## Abstract

Spain exhibits significant climatic variability across its regions, ranging from the humid oceanic climate in the north to the Mediterranean and stepped climates in the southern and central areas. These climatic differences influence environmental allergen exposure, which may, in turn, affect allergic sensitization patterns in the pediatric population. Variations in temperature, humidity, and airborne allergen distribution, such as pollen, dust mites, animal epithelia, and mold, contribute to regional disparities in allergic responses among children. Understanding how climatic conditions shape allergen recognition patterns across different geographical areas is essential for improving allergy prevention and management strategies. This study aims to shed light on this issue by identifying sensitization patterns in a pediatric population of 236 patients (with 2 age groups analyzed: 6–10 and 11–15 years old) from different climatic regions of Spain. Skin tests with standard aeroallergens were performed, and specific IgE (sIgE) analysis by Allergy Explorer of Macroarray Diagnostic test (ALEX^2^) and Western blot. The results revealed different sensitization trends across patients from the eight cities analyzed (Barcelona, Granada, Lugo, Sagunto, Santiago de Compostela, Valencia, and Zaragoza).

## 1. Introduction

Allergic diseases, including asthma and allergic rhinitis, are increasingly prevalent in pediatric populations worldwide and represent a significant burden on healthcare systems and quality of life [1,2]. The development of allergic sensitization is largely driven by exposure to aeroallergens, such as house dust mites, pollens, fungi, and animal epithelia [3]. However, sensitization patterns are not uniform across populations, as they are influenced by multiple environmental and demographic factors [4,5,6]. Understanding these variations is essential to improve diagnostic strategies, preventive measures, and targeted treatments for allergic diseases in children [7].

Geographical and climatic conditions play a crucial role in shaping regional allergen exposure. Dust mites and fungi thrive in humid environments, increasing the risk of sensitization to these allergens [8,9]. Conversely, regions with higher temperatures and lower humidity levels tend to have higher pollen concentrations and longer pollen seasons, leading to increased sensitization rates [10,11]. In addition, urbanization and air pollution have been identified as major environmental contributors to allergic diseases, as they can increase allergenicity and alter immune responses in predisposed individuals [3,12].

Beyond environmental factors, demographic variables, such as age, sex, and genetic background, also influence allergic sensitization patterns [13,14,15]. Previous studies have suggested that younger children may have a higher prevalence of sensitization to indoor allergens, such as house dust mites, whereas older children and adolescents show increasing sensitization to outdoor allergens, such as pollen [16,17]. The transition from childhood to adolescence is also associated with maturation of the immune system, which may influence IgE reactivity and allergic responses [18].

Given these complex interactions, comparative studies analyzing aeroallergen sensitization across different regions are needed to identify risk factors and improve disease management [19]. In Spain, differences in climate, biodiversity, and urbanization levels between different cities provide an opportunity to study regional differences in allergic sensitization in pediatric populations [20]. However, few studies have systematically analyzed these differences, especially in children from different geographical areas [21].

The aim of this study was to compare aeroallergen sensitization patterns in pediatric populations from different Spanish cities, including Granada, Madrid, Valencia, Sagunto, Santiago, Lugo, Zaragoza, and Barcelona. By analyzing 2 different age groups (4–10 years and 11–15 years old, y.o.), we aim to investigate how geographical and demographic factors influence allergic sensitization [22,23,24]. The results may provide valuable insights into the development of pediatric allergies and contribute to more personalized approaches to allergy diagnosis and treatment in Spain.

## 2. Results

### 2.1. Demographic Features of Investigated Patients

All 236 subjects (an average of 30 patients per center, 8 centers in total) who met the ARIA or GINA criteria for allergic rhinitis (AR) and/or asthma (A) [25,26] tested positive for one or more aeroallergens in a skin prick test (SPT). Most participants were male (62.7%) with a median age of 10.0 years (range: 4–15) (Table 1).

None of the patients had received allergen immunotherapy or biologic treatments before or during the study inclusion. Atopic comorbidities were present, with food allergies (to seafood, fruit, legumes, nuts, eggs, and/or milk) affecting 22.88% (54 patients).

### 2.2. Prevalence, sIgE Reactivity and Individual Molecular Profile According to Atopic Disease

The sensitization to aeroallergen extracts through SPT and the prevalence of the 236 patients who met the inclusion criteria are summarized in Table 2.

**Figure 1:**
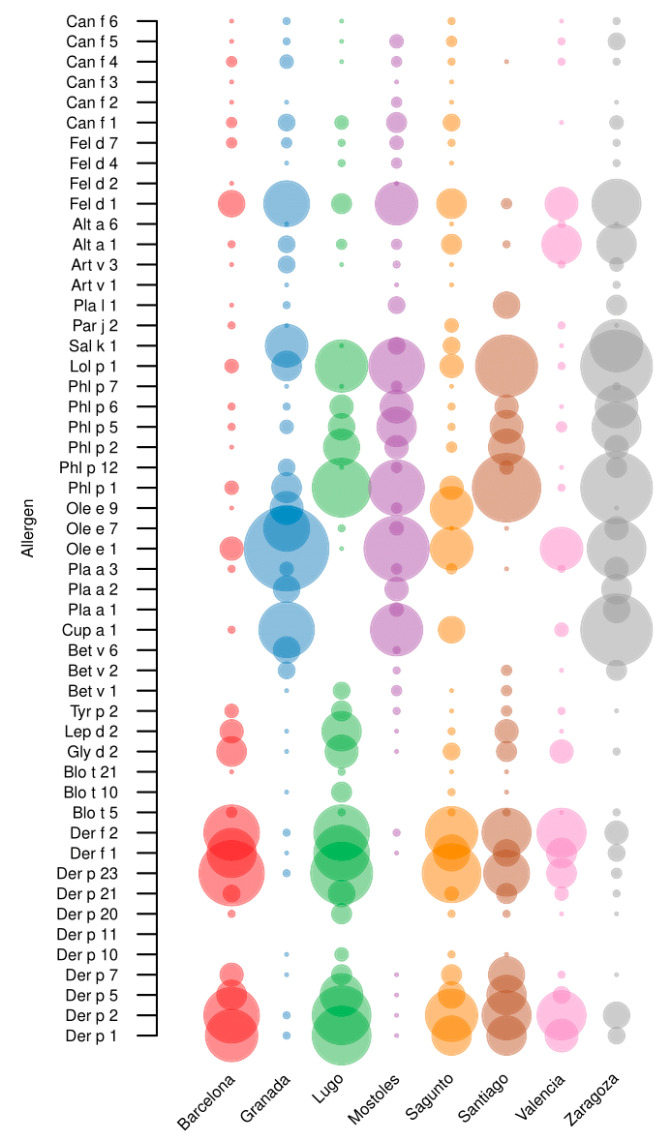
Bubble plot illustrating the distribution of airborne allergens in each region. The size of the bubbles is directly proportional to the number of patients who are sensitized (sIgE ≥ 0.3 kU_A_/L) to the allergen.

Overall, 231 out of 236 patients (97.88%) were sIgE-positive (≥0.35 kU_A_/L) to 1 or more of the 52 individual molecular aeroallergens included in the multiplex array (Figure 1).

Despite having a positive SPT for at least one of the investigated aeroallergens, 4 patients (1.69%) did not display sIgE levels above 0.35 kU_A_/L for any of the allergens tested in the multiplex array.

### 2.3. Mites

Mites have been identified as the predominant source of sensitizing airborne allergens in the cities of Barcelona, Lugo, Sagunto, Santiago, and Valencia (Figure 1). Sensitization to one or more of the 17 mite molecular allergens studied was observed in 125 subjects, representing 53.0% of the study population. In addition, in the 5 centers where mites were most prevalent, 73.5% (108 patients) had positive sIgE values for some mite molecular allergens. Der p 1, Der p 2, Der f 1, Der f 2, and Der p 23 were all identified by our results as major allergens (prevalence above 55%) in this group, with Der p 2 being the most recognized. Der p 5, Der p 7, Der p 21, Gly d 2, and Lep d 2 were mid-tier allergens, with a prevalence of 20% to 40% (Table 3). Minor allergens, with a prevalence of less than 20%, were Tyr p 2, Der p 10, Der p 20, Blo t 5, Blo t 10, and Blo t 21. However, no patient recognized Der p 11.

The overall prevalence of subjects exhibiting sIgE to group 1 allergens—Der p 1 and Der f 1—was recorded at 61.6%, which is lower than the prevalence observed for group 2 allergens—Der p 2 and Der f 2—at 75.5%, as well as for Der p 23, which stood at 74.1%. Among the individual molecular allergens, Der p 2 was identified most frequently, with a prevalence of 75.9%. This was followed closely by Der f 2 at 75.0%, Der p 23 at 74.1%, Der p 1 at 64.8%, and Der f 1 at 58.3%. Among the subjects sensitized to group 1 allergens (61.6%), a significant majority (90.0%; 63 out of 70 subjects) exhibited positive reactions for both Der f 1 and Der p 1. Notably, 8 subjects were exclusively sensitized to Der p 1, while one subject demonstrated sensitivity solely to Der f 1. In a similar vein, 97.6% of subjects sensitized to group 2 allergens exhibited sIgE to both Der f 2 and Der p 2 (2 subjects were exclusively sensitized to Der p 2, and 1 subject was exclusively sIgE to Der f 2).

Most subjects (88.8%) recognized between 1 and 9 of the tested mite allergens tested. The most common sIgE profile, consisting of two molecules, included any of the five major allergens. Sensitization to more than 9 mite allergens was rare (11.2%). The profile of mono-sensitized individuals is also very diverse: only 1 out of 125 patients (0.8%) showed exclusive sensitization to Der p 7; 2 patients (1.6%) displayed sensitization to Der p 20; 6 patients (4.8%) recognized Der p 23; 1 patient (0.8%) was sensitized only to Der f 1 and also 1 (0.8%) to Der f 2; 2 patients (1.6%) recognized only Lep d 2; and 1 patient (0.8%) to Blo t 5.

#### 2.3.1. sIgE Reactivity Profiles by Geographic Location

**Figure 2:**
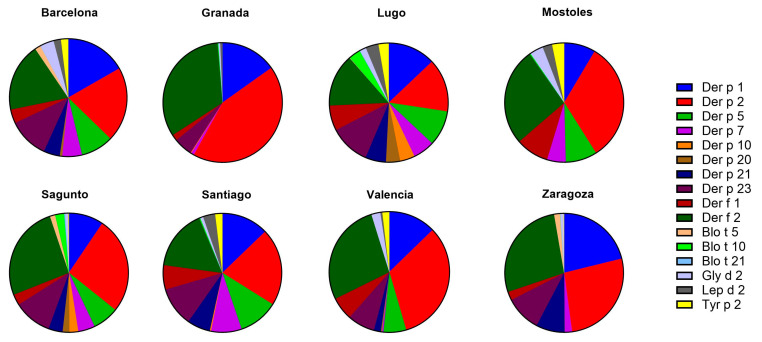
Mean levels of sIgE for each mite allergen among all patients from eight different populations in Spain (Barcelona, Granada, Lugo, Mostoles, Sagunto, Santiago, Valencia, and Zaragoza). Each pie chart represents the proportional distribution of sIgE reactivity to different allergens, as indicated by the color-coded legend. Allergen abbreviations correspond to *Dermatophagoides pteronyssinus* (Der p) and *Dermatophagoides farinae* (Der f) allergens, as well as other mite species (*Blomia tropicalis*, *Glycyphagus domesticus*, *Lepidoglyphus destructor*, and *Tyrophagus putrescentiae*).

Among the analyzed mite allergens, the most relevant ones, based on higher sIgE levels and sensitization frequency, were Der p 1, Der p 2, Der p 23, Der f 1, and Der f 2, followed by Der p 5, Der p 7, Der p 21, Gly d 2, Lep d 2, and Tyr p 2 with lower titers and frequencies (Figure 2). All centers had one or more positive patients for these major allergens—Der p 1, Der p 2, Der f 1, Der f 2, and Der p 23—except for Mostoles, with no patients sensitized to Der p 23. Der p 1 is the most predominant mite allergen in Lugo (60.0%), while Der p 2 exhibited the highest frequency in Valencia (50.0%) and Zaragoza (26.7%). Der p 23 was the most prevalent in Barcelona (74.1%), Santiago (66.7%), and Sagunto (60.0%), whereas Der f 2 was also highly recognized in Valencia (50.0%). Although Der f 1 showed a high sensitization rate, it was not the most frequent mite allergen in any of the centers included in this study.

Variations in mite allergen recognition pattern across the different regions were also observed. In Barcelona, Lugo, and Santiago de Compostela, there was a high recognition of all previously mentioned key allergens (*Dermatophagoides* family, *Glycyphagus*, *Lepidoglyphus*, and *Tyrophagus*). Additionally, in Sagunto, Valencia, and Zaragoza, a strong recognition of allergens from the *Dermatophagoides* family was evident. In contrast, Granada and Mostoles showed a markedly lower number of patients sensitized to mite allergens (Table 3).

For *Blomia tropicalis*, minimal sensitization was observed, with Blo t 10 being the most strongly allergen recognized from this source in Lugo.

#### 2.3.2. Age and sIgE Reactivity Profiles

In Barcelona, Lugo, Sagunto, and Valencia, younger patients (<11 y.o., n = 84) displayed a higher frequency of sIgE binding to 6 out of 17 molecular mite allergens—including the five major allergens (Der p 1, Der p 2, Der f 1, Der f 2, and Der p 23) and any of the five mid-tier allergens (Der p 5, Der p 7, Der p 21, Gly d 2, and Lep d 2)—compared to older patients (≥11 y.o., n = 63). In addition, the percentage of patients in group 1 (<11 y.o.) who recognized any of the mite molecular allergens was higher—94.4% in Barcelona, 100% in Lugo, and 73.3% in Sagunto and Valencia—than in group 2 (≥11 y.o.), except for Santiago de Compostela (46.7% of the patients in group 1 and 80% of the patients in group 2). Moreover, in the Santiago de Compostela cohort, older subjects exhibited a higher frequency of sensitization to more than 9 mite molecular allergens compared to those that were younger.

### 2.4. Pollens

Pollen plays a significant role in the studied areas, being the most relevant allergen in the cities of Granada, Mostoles, and Zaragoza (Figure 1). A similar analysis was conducted for molecular components of pollen. In this case, sensitization to one or more allergens was observed in 170 out of 234 patients (72.7%). This frequency increased when considering only the centers where a pollen allergy was highly significant (93.3%). In this population, the molecules that were considered major allergens (>50% sensitized individuals) were Cup a 1, Ole e 1, Phl p 1, and Lol p 1. Mid-tier allergens, with a prevalence of 20 to 40%, were Pla a 2, Ole e 7, Phl p 5, Phl p 6, and Sal k 1. Lastly, minor allergens (prevalence less than 20%) were Bet v 1, Bet v 2, Bet v 6, Pla a 1, Pla a 3, Ole e 9, Phl p 2, Phl p 7, Phl p 12, Par j 2, Pla l 1, Art v 1, and Art v 3.

When analyzing pollen allergens by plant family, distinct patterns emerged across the studied populations. Among *Oleaceae*, Ole e 1 was the most relevant (71.9%), while in grasses (*Poaceae*), Phl p 1 and Lol p 1 were the predominant sensitizing molecules (53.9%). For Betulaceae, Bet v 2 exhibited the highest recognition (14.6%), whereas in *Platanaceae*, Pla a 2 was the most frequently detected (26.9%). Finally, among *Asteraceae*, Art v 3 (12.4%) stood out as the most significant.

Most subjects (82.0%) recognized between 1 and 9 of the pollen allergens analyzed. The most frequently observed sIgE profile involved five allergens, including any of the four major allergens—Cup a 1, Ole e 1, Phl p 1, and Lol p 1. Sensitization to more than 9 pollen allergens was observed in a limited number of subjects (11.2%). Mono-sensitization patterns were very similar, with 3 subjects that only recognized Ole e 1 (3.4%) and 2 patients exhibiting exclusive sensitization to Cup a 1 (2.2%). Only one monosensitized patient recognized a non-major allergen, Phl p 5 (1.1%).

#### 2.4.1. sIgE Reactivity Profiles by Geographic Location

**Figure 3:**
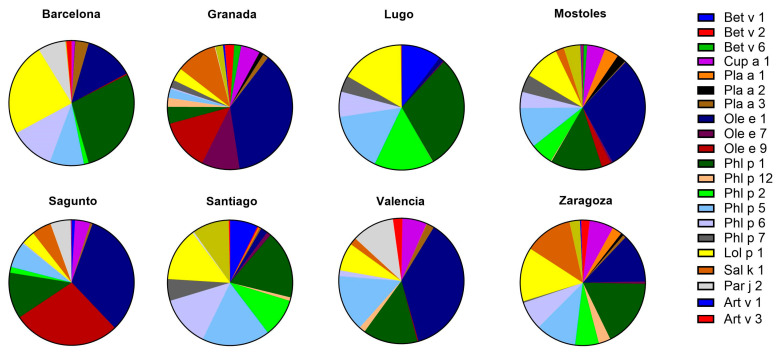
Mean levels of sIgE illustrating sensitization to different pollen allergens in patients from eight populations in Spain (Barcelona, Granada, Lugo, Mostoles, Sagunto, Santiago, Valencia, and Zaragoza). Each pie chart represents the relative distribution of sIgE against various pollen allergens, according to the color-coded legend. Sensitizations include allergens from *Betula* (Bet v 1, Bet v 2, Bet v 6), *Cupressus* (Cup a 1), *Platanus* (Pla a 1, Pla a 2, Pla a 3), *Olea* (Ole e 1, Ole e 7, Ole e 9), *Phleum* (Phl p 1, Phl p 12, Phl p 2, Phl p 5, Phl p 6, Phl p 7), *Lolium* (Lol p 1), *Salsola* (Sal k 1), *Parietaria* (Par j 2), and *Artemisia* (Art v 1, Art v 3).

When analyzing the cities separately, it was observed that in Barcelona the most prevalent allergen was Ole e 1, with 7 sensitized patients (25.9%). In Granada, there was a broad recognition of multiple pollen allergens, with Ole e 1 again being the most frequent, affecting 26 patients (86.7%). Similarly to Granada, individuals in Mostoles exhibited a wide range of sensitization to various allergens, with Ole e 1 being the most prevalent (69.0%). In Sagunto, Ole e 1 and Ole e 9 were recognized by 13 patients (43.3%), whereas in Valencia, Ole e 1 remained the most frequently detected allergen, with 13 patients (43.3%), despite the overall lower recognition of pollen allergens in this area compared to the other centers. Lugo and Santiago de Compostela (North Spain) exhibited a highly similar pattern of pollen allergen recognition, with comparable sIgE titers and sensitization frequencies. In both cities, Phl p 1 was the most prevalent allergen, with 18 sensitized patients in Lugo (60.0%) and 13 in Santiago (43.3%). Finally, in Zaragoza, a widespread recognition of pollen allergens was evident, with a high number of patients sensitized to most of the tested molecules. Among them, Cup a 1, Phl p 1, and Lol p 1 stood out due to their higher prevalence, each being recognized by 22 out of 30 patients included in this area (73.3%) (Figure 3 and Table 4).

#### 2.4.2. Age and sIgE Reactivity Profiles

Mainly no differences in the prevalence of pollen sensitization were observed between the two age groups studied, with 85 (36.0%) younger patients (<11 y.o.) recognizing at least one pollen molecular allergen (sIgE ≥ 0.3 kU_A_/L), and the same number of patients in group 2 (≥11 y.o.). Although in Granada, Mostoles and Zaragoza, older patients (≥11 y.o.) exhibited sIgE sensitization profiles with the highest number of molecular pollen allergens (more than 10 out of 22) at the same time. This type of sensitization profile included the four major allergens (Cup a 1, Ole e 1, Phl p 1, and Lol p 1) and any of the five mid-tier allergens (Pla a 2, Ole e 7, Phl p 5, Phl p 6, and Sal k 1). It should be noted that the prevalence of Sal k 1 was approximately double in the older group (≥11 y.o.) compared to the younger group (27.3 vs. 48.9%), and the same for Pla a 1 (9.1 vs. 17.8%), Ole e 7 (20.5 vs. 35.6%), Ole e 9 (11.4 vs. 20.0%), Phl p 12 (11.4 vs. 20.0%), and Phl p 7 (4.5 vs. 8.9%). In addition, for Pla l 3, it was three times higher (6.8 vs. 22.2%). Furthermore, Art v 1 was not recognized by any patient in the first group, but its prevalence in the second group was 8.9%.

### 2.5. Cat and Dog Epithelia

The distribution of sensitization to dog and cat epithelial allergens showed slight variation across the eight analyzed regions. Among the 236 patients, 104 tested positive for 1 or more allergens from these sources (44.0%). Of those sensitized, 64 were mono-sensitized to a single allergen (61.5%). In contrast, 2 patients (1.9%) exhibited recognition of 8 out of the 10 animal epithelial allergens studied.

Fel d 1 was established as the only major allergen form this group (80.8%). In dog dander, the most recognized allergen was Can f 1 (28.8%).

### 2.6. Molds

Overall, molds were not an important allergen source in the study population (Figure 1), since only 45 out of 236 patients (19.1%) were sensitized to any of the fungi molecular allergens investigated. In fact, 44 of these patients (97.8%) showed positive sIgE (sIgE ≥ 0.3 kUA/L) to Alt a 1, so it could be considered a major allergen in fungi. Moreover, 4 out of 45 patients were polysensitized to both *Alternaria* allergens (Alt a 1 and Alt a 6). Furthermore, only one subject belonging to the Sagunto cohort was monosensitized to Alt a 6.

Although the general prevalence of mold was low, in Valencia, Sagunto and Zaragoza, it played an important role. Specifically, in Valencia and Zaragoza, 40.0% of patients were sensitized to fungi, while in Sagunto, it was 23.3%.

### 2.7. IgE Western Blot

**Figure 4:**
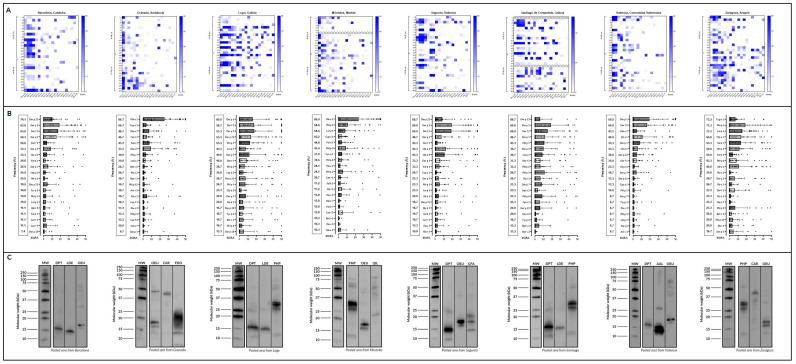
Sensitization profile to specific IgE (sIgE): (**A**) heatmap and (**B**) scatter plots with bars of the 20 most frequently identified molecular allergens in each region, along with their serodominance. (**C**) IgE-Western blot of the different groups included against the three most prevalent allergen sources in each region. Different patterns of sIgE binding were identified for each group of patients.

Western blot analysis was carried out for each geographic cohort to study different allergenic profiles from the three most prevalent allergen sources (Figure 4C). In that way, *Dermatophagoides pteronnysinus* profile showed a 16-kDa prominent band and 2 others (11 and 26 kDa) with less intensity in Barcelona. However, a 14-kDa protein band and a less intense 25-kDa protein band were observed in *Lepidoglyphus destructor* for the same population. Moreover, for olive pollen we observed 2 bands between 15 and 20-kDa that could be Ole e 1. Also, Ole e 9 appeared like a higher molecular weight band (46 kDa).

Granada was noted for the high prevalence of olive pollen, as well as pollen of *Cupressaceae*. The olive allergenic profile showed three notable protein bands that could correspond to the most prevalent olive pollen allergens (Ole e 1 and Ole e 9), according to the molecular results cited above. While for *Cupressaceae*, we studied *Cupressus arizonica* pollen, and our immunoblot analysis showed a ~43 kDa protein band, corresponding to Cup a 1 (pectate lyase), the major allergen from these sources. Moreover, cat dander was important in this cohort, highlighting an intense ~20 kDa protein which could be Fel d 1. 

In Mostoles, immunoblot results showed three intense protein bands between 25 and 37 kDa in *Phleum pratense*, which could include the major allergen Phl p 1, and Phl p 5. There were also other protein bands of lower (~12–13 kDa) and higher (50–75 kDa) molecular weight, probably related to any mid-tier and/or minor allergens. In addition, two intense protein bands were detected in olive pollen, presumably Ole e 1 (major allergen), and Ole e 9 at 45 kDa. Although no allergens from *Quercus ilex* were included in ALEX^2^, we ran its allergen profile due to its high positivity by skin test, and a 23-kDa protein band was clearly observed.

Dust mites were the most prevalent source in Galicia (Lugo and Santiago), so we analyzed their allergenic profiles. In both populations, the ~16-kDa band was the most prominent in *D. pteronyssinus*, belonging to Der p 2, but there were other bands less prominent at 12, 14, and 25 kDa related to other major allergens, like Der p 1 and Der p 23. This similarity between populations was also observed for *L. destructor*, with two prominent ~15 and ~25 kDa bands. In terms of pollen, we performed *Phleum* allergenic profile analysis on both populations, with similar results observed for Mostoles.

Furthermore, in Sagunto, we performed the allergenic profile from dog dander. Immunoblot results showed 3 prominent bands (14, 17, and 21 kDa) due to sIgE sensitization to lipocalins as Can f 1, the major allergen in *Canis familiaris*. On olive pollen, 2 bands below 20-kDa, likely Ole e 1, were highlighted. In *D. pteronyssinus*, intense sensitization to Der p 2 was shown as a 15-kDa protein band.

Molds had importance in Valencia, with 2 notable protein bands of 13 and 14 kDa in *Alternaria alternata* that could be Alt a 1, according to the sIgE results obtained. Furthermore, a 27-kDa protein band appeared, it could probably correspond to an allergen that was not included in the microarrays. On olive pollen, Ole e 1 was predominantly shown for this population, but it was interesting to recognize several high molecular weight bands in contrast to the rest of the olive profiles.

Finally, the Zaragoza blot showed the same allergen profile in grass pollen as in the other regions of Spain where this source is very prevalent, such as Mostoles, highlighting bands compatible with Phl p 1 and Phl p 5. Also, Western blot results suggested that Ole e 1 and Cup a 1 would be the most recognized allergens in olive and cypress pollen, respectively.

## 3. Discussion

The result of our study reveals heterogeneous patterns of aeroallergen sensitization in children across different regions of Spain (Barcelona, Granada, Lugo, Mostoles, Santiago, Sagunto, Valencia, and Zaragoza), influenced by environmental factors, climate conditions, and urbanization levels (Figure 4). This highlights the need for region-specific diagnostic and therapeutic strategies. Additionally, the findings support previous research linking the prevalence of allergic diseases to temperature, humidity, and pollution, as well as to diet and microbiota composition, demonstrating a complex interaction between genetic predisposition and environmental factors [19,22,23].

**Figure 5:**
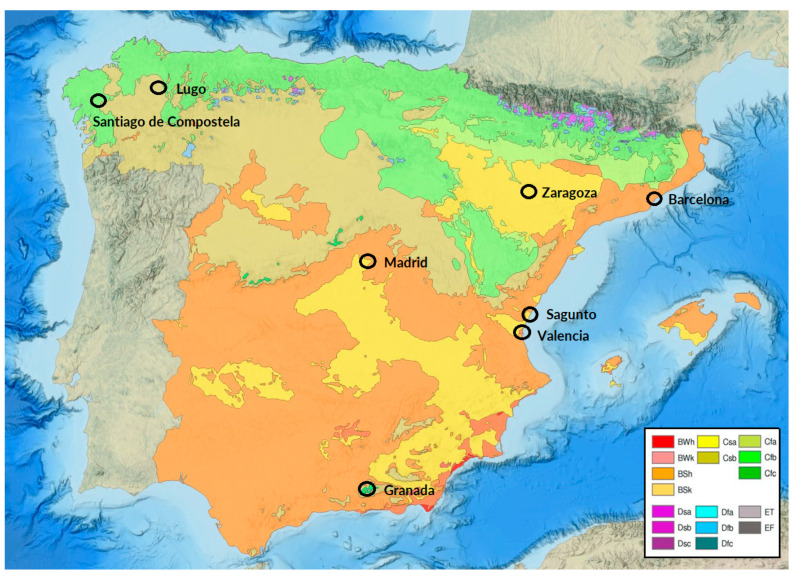
Köppen–Geiger climate map of research centers. Modified figure of climate classification map according to Köppen from the National Atlas of Spain (ANE) [24].

Spain’s climate varies significantly due to its diverse geography, according to the Köppen–Geiger classification [20]. Santiago de Compostela and Lugo are located in Galicia and they have an oceanic climate (Cfb) with mild temperatures and high rainfall due to Atlantic influences. Zaragoza, with a steppe or cold semi-arid climate (BSk), experiences extreme temperature variations and low precipitation due to its inland location. Madrid and Barcelona share a hot-summer Mediterranean climate (Csa), but Barcelona’s maritime influence moderates seasonal changes. Valencia and Sagunto also have a Csa climate, with dry summers and mild, wetted winters. Granada transitions between Csa and BSk climates, with hot summers, cold winters, and low precipitation. These classifications highlight Spain’s climatic diversity shaped by geographical and atmospheric factors (Figure 5).

Air quality significantly impacts aeroallergen concentration and distribution in urban and industrial areas. Despite improvements in some cities, pollution remains a concern. Barcelona has reduced NO_2_ levels by 43% in the last decade due to low emission zones, yet PM_10_ and PM_2.5_ still exceed WHO limits [25], particularly in high-traffic areas. Granada continues to record high NO_2_ concentrations from traffic and industrial PM_10_ emissions, while Lugo experiences SO_2_ exceedances from cement production, potentially increasing pollen allergenicity [26].

Santiago de Compostela shows PM_10_ and PM_2.5_ levels above WHO guidelines [25], and Sagunto’s industrial activity results in high ozone, PM, and NO_2_ levels, lacking an air quality improvement plan. Mostoles, influenced by Madrid’s pollution, likely has high NO_2_ and PM levels. In Valencia, NO_2_, PM_10_, and ozone levels surpass WHO standards, affecting 47% of the population, while Zaragoza’s traffic and industry likely contribute to high NO_2_ and PM concentrations [26].

Overall, urban traffic and industry drive poor air quality, exacerbating allergic reactions [26]. Stricter policies are needed to mitigate exposure to these pollutants and reduce health risks [27].

Our findings confirm that mites are the predominant airborne allergens in several Spanish cities, with a sensitization rate of 53.0% in the study population [28,29]. The prevalence was even higher in the five examined regions with the most humid climates (Barcelona, Lugo, Santiago, Sagunto, and Valencia), with a rate of 73.5%. Among the 17 mite molecular allergens analyzed, Der p 1, Der p 2, Der f 1, Der f 2, and Der p 23 were identified as the major allergens, with Der p 2 being the most frequently recognized (75.9%). Mid-tier allergens, such as Der p 5, Der p 7, and Lep d 2, showed intermediate prevalence (20–40%), while minor allergens had a prevalence below 20%. Notably, no patients recognized Der p 11, demonstrating the low prevalence of this allergen in patients with rhinitis and/or asthma [30,31].

In that way, our data indicate higher sensitization rate to group 2 allergens (75.5%) compared to group 1 (61.6%), confirming their importance in allergic disease development [32]. This aligns with previous epidemiological studies demonstrating that dust mites are the leading aeroallergen in humid climates and indoor environments [8,19].

Age-related differences were also significant [33]. Younger children (6–10 years) showed higher sensitization rates to Der p 1 and Der p 2 than adolescents (11–15 years), suggesting that early mite exposure may shape allergic sensitization and disease progression [34]. Immune immaturity in younger children likely enhances Th2 responses, promoting IgE-mediated allergies [35]. Over time, immune regulation and microbial exposure may modify sensitization patterns in older children [36,37,38].

Pollen sensitization was highly variable across Spain, with an overall prevalence of 72.7%. However, the highest rates were observed in Zaragoza, Granada, and Mostoles. First, in Zaragoza, Phl p 1 (timothy grass), Lol p 1 (ryegrass), and Sal k 1 (tumbleweed) were predominant allergens [39]. Similarly, significant differences were observed in Ole e 1 (olive pollen) and Cup a 1 (cypress pollen) sensitization rates, which were highest in Granada and Zaragoza, respectively. This supports previous research emphasizing the influence of regional vegetation and agricultural activities on airborne pollen levels, underscoring the need to integrate geographical factors into allergy diagnosis and treatment planning [40].

Most individuals (82.0%) recognize 1–9 allergens, typically including a major one. Highly polysensitized cases (>9 allergens) are rare (11.2%), and mono-sensitization is uncommon, mainly to Ole e 1 (3.4%), Cup a 1 (2.2%), and Phl p 5 (1.1%). No significant differences in overall pollen sensitization prevalence were observed between age groups, with 85 younger (<11 y.o.) and 85 older (≥11 y.o.) patients testing positive (36.0% each). However, in Granada, Mostoles, and Zaragoza, older patients showed broader sensitization profiles, frequently recognizing more than 10 allergens, including the 4 major ones and mid-tier allergens, such as Pla a 2, Ole e 7, Phl p 5, Phl p 6, and Sal k 1. Some allergens were notably more prevalent in the older group, such as Sal k 1 (27.3% vs. 48.9%), Pla a 1 (9.1% vs. 17.8%), Ole e 7 (20.5% vs. 35.6%), Ole e 9 (11.4% vs. 20.0%), Phl p 12 (11.4% vs. 20.0%), and Phl p 7 (4.5% vs. 8.9%). The prevalence of Pla l 3 was three times higher (6.8% vs. 22.2%) in older individuals, and Art v 1, absent in younger patients, reached 8.9% in the older group age, reinforcing the need for age-adapted diagnostic and therapeutic approaches [41].

Sensitization to mold allergens was relatively uncommon, affecting only 19.1% of the study population, although regional variations in prevalence were observed. This low prevalence is consistent with previous epidemiological studies, which indicate that fungal allergens generally play a secondary role in allergic sensitization compared to pollen and dust mites. However, in specific regions, such as Valencia, Sagunto, and Zaragoza, fungal sensitization was notably higher. From this approach, almost 50% of the studied population in Zaragoza were surprisingly sensitized to *Alternaria alternata*. This could be due to its dry and hot climate, which favors the proliferation of molds, and to the high concentration of spores in the air, especially in summer and autumn [9]. In addition, the ‘Cierzo’ winds contribute to its dispersion and to the increase of allergic reactions [42].

Alt a 1 emerged as the predominant allergen, recognized by 97.8% of sensitized individuals, reinforcing its status as a major *Alternaria* allergen [43]. This aligns with prior studies demonstrating that Alt a 1 is the primary target of IgE responses in *Alternaria*-sensitized individuals and is strongly associated with allergic rhinitis and asthma [44]. Furthermore, a small group of patients (4/45, 8.9%) exhibited polysensitization to both Alt a 1 and Alt a 6, which may indicate broader immune recognition of *Alternaria* components in these individuals. Interestingly, only one patient from the Sagunto cohort was monosensitized to Alt a 6, suggesting that this allergen plays a more limited role in fungal sensitization [45].

The observed regional differences in fungal sensitization rates could be attributed to climatic and environmental conditions that favor fungal spore proliferation, particularly in areas with high humidity and mild temperatures [9,43,44]. Further investigation into local environmental factors, fungal spore concentrations, and patient symptomatology could provide valuable insights into the clinical relevance of fungal allergens in these regions.

The observed distribution of sensitization to cat and dog epithelial allergens across the eight analyzed regions suggests a relatively high prevalence of animal epithelium-related allergies, affecting 44.0% of the studied population. This frequency is consistent with previous epidemiological studies, which highlight pet allergens as common triggers of allergic diseases, particularly in urban environments where pet ownership is widespread [46].

Among sensitized individuals, the predominance of monosensitization (61.5%) suggests that exposure to a single dominant allergen is sufficient to elicit an immune response in most cases. However, there was a small subset of polysensitized patients (1.9%) who may have a heightened immune reactivity or broader cross-reactivity patterns [47,48]. This group warrants further investigation, as polysensitization is often associated with more severe allergic manifestations and increased likelihood of comorbidities, such as asthma or atopic dermatitis [49].

The identification of Fel d 1 as the primary allergen in cat dander (80.8%) aligns with its well-documented role as the major sensitizer among cat-allergic individuals. Given that Fel d 1 is a highly diffusible protein, it is known for its persistence in the environment, even in spaces where cats are not present [50]. This reinforces its clinical relevance in allergic disease management, particularly in cases of indirect exposure [48].

In contrast, sensitization to dog allergens appears to be more heterogeneous, with Can f 1 emerging as the most recognized component (28.8%) [51]. The lower percentage compared to Fel d 1 may reflect differences in the allergenic potential of dog-derived proteins, variations in exposure levels, or differing immune response mechanisms between dog and cat allergens [52]. Additionally, given the existence of multiple clinically relevant dog allergens (e.g., Can f 2, Can f 3, Can f 5), further stratification of sensitized individuals by their specific IgE reactivity profiles could provide a more nuanced understanding of dog allergy patterns [52,53,54].

In contrast to the study by González et al. [19], which compared sensitization patterns in two adult populations with similar climatic conditions, our study analyzed the geographical variability of sensitization in a pediatric cohort distributed across eight centers with diverse climates and environmental exposures. While González et al. [19] focused on common immunological mechanisms in populations with similar climatological conditions, emphasizing the importance of mites in tropical regions, our work aimed to characterize how specific geographical factors influence sensitization to aeroallergens. Thus, our study provides a broader perspective on the distribution of allergies in Spain.

In comparison to the study by Yoon et al. [55], which investigated the molecular mechanisms underlying aeroallergen sensitization and allergic rhinitis through transcriptomic analysis of nasal brushings, our study focuses on sensitization to common allergens (mites, pollens, fungi, and epithelia) by measuring sIgE. Both studies agree that mites and pollens are the most prevalent allergens, with a sensitization prevalence of 74.5% and 57.4%, respectively, in our case. However, Yoon et al.’s approach allows for a more detailed analysis of the molecular mechanisms through causal network analysis, identifying key subnetworks related to lymphocyte chemotaxis and mast cells, while our study focuses on the prevalence and age-related differences in sensitization to these allergens, providing a more clinical and demographic perspective. Both studies, although with different methodologies, highlight the complexity of sensitization and its biological foundations, contributing to the understanding of the immune mechanisms involved in allergic rhinitis.

This analysis highlights how different climatic conditions influence the dominance of allergenic sources across Spain. Regions with high humidity, such as coastal and oceanic areas, see an increased prevalence of dust mites and fungal spores, while dry and semi-arid areas foster pollen from grasses and drought-resistant plants [9,56,57]. The Mediterranean climate supports a variety of tree pollens, such as olive, cypress, and plane tree, which significantly impact seasonal allergy sufferers [58,59]. Understanding these climatic correlations allows for improved allergen forecasting and more targeted preventive measures for allergic individuals.

The findings of this study pave the way for future research in several key areas. However, it is important to highlight a limitation of this study, the lack of data on infants (0–5 y.o.) due to the difficulty of recruiting patients at such an early age. Consequently, we were unable to analyze the onset of allergies. This is primarily because many children have not yet exhibited allergic symptoms at such an early stage. Future research that includes this age group is necessary to overcome this limitation. Also, the study could be expanded into a longitudinal design to analyze the progression of allergies and symptoms over time. This would allow for the examination of differences in the impact of age within the same individuals.

Additionally, the role of environmental pollutants and microbiome alterations in modulating allergic responses warrants further investigation [60]. Emerging research suggests that air pollution, particularly fine particulate matter, and nitrogen oxides, may exacerbate allergic inflammation and increase allergen sensitization rates [2,12,61]. Understanding these interactions will be crucial for developing comprehensive public health strategies to mitigate allergy burden in pediatric populations.

Moreover, advances in precision medicine, including genetic and epigenetic analyses, may provide novel insights into individual susceptibility to aeroallergens and guide the development of more personalized treatment approaches [41,61].

## 4. Materials and Methods

### 4.1. Subjects

Between December 2023 and January 2025, we consecutively recruited children aged 5 to 15 years (divided into 5-year age groups) with a confirmed diagnosis of allergic rhinitis and/or asthma from hospitals across different climatic regions of Spain [20]. This investigation was reviewed and approved by the Ethical Committee of each center, and informed consent was obtained from all participants and from parents/guardians at the time of their inclusion in the study. The severity and stage of allergic diseases were clinically assessed following specific guidelines.

Clinical data collected from patients’ medical records included sociodemographic information, medical history (including past medical conditions and current allergy diagnosis), and details about their treatments. In line with routine clinical practice, only patients with a positive skin prick test (SPT) to relevant aeroallergen extracts (such as mites, pollens, molds, and animal epithelia) were included in the study. Patients who had received or were currently undergoing allergen immunotherapy or treatment with monoclonal antibodies (biologics) were excluded.

### 4.2. Skin Prick Test

Percutaneous testing was conducted according to European standards using a diagnostic panel (Inmunotek S.L., Madrid, Spain) with standardized extracts, including *Dermatophagoides pteronyssinus* (*D. pteronyssinus*), *Blomia tropicalis* (*B. tropicalis*), *Lepidoglyphus destructor* (*L. destructor*), cat and dog dander, a grass mix (*Poa pratensis*, *Dactylis glomerata*, *Lolium perenne*, *Phleum pratense*, and *Festuca pratensis*), *Olea europaea*, *Betula verrucosa*, *Quercus ilex*, *Platanus hispanica*, *Plantago lanceolata*, *Parietaria judaica*, *Salsola kali*, *Artemisia vulgaris*, *Juniperus oxycedrus*, *Cupressus arizonica*, *Cupressus sempervirens*, and *Alternaria alternata* [62,63]. Histamine (10 mg/mL) and saline were used as positive and negative controls, respectively. As per standard procedure, antihistamines were discontinued one week before the SPT. Wheal diameters were measured 20 min after testing, with diameters greater than 3 mm considered positive [64].

### 4.3. Serological Analysis

Blood samples were collected from all participants, labeled with a unique code, stored at −40 °C, and thawed immediately before in vitro testing. Total IgE and specific IgE (sIgE) levels were measured using the ALEX MacroArray platform (MacroArray Diagnostics, Vienna, Austria) [65] according to the manufacturer’s protocol. ALEX is a multiplex array containing 295 reagents, including 117 whole allergens and 178 molecular components. The allergens were attached to polystyrene nanobeads, which were then deposited on a nitrocellulose membrane, as previously described [66]. The assay included 17 mite molecular allergens: Der p 1, Der p 2, Der p 5, Der p 7, Der p 10, Der p 11, Der p 20, Der p 21, Der p 23, Der f 1, Der f 2, Blo t 5, Blo t 10, Blo t 21, Lep d 2, Gly d 2, and Tyr p 2. Additionally, 10 cat and dog epithelial allergens were tested: Fel d 1, Fel d 2, Fel d 4, Fel d 7, Can f 1, Can f 2, Can f 3, Can f 4, Can f 5, and Can f 6. A total of 22 pollen allergens were included: Bet v 1, Bet v 2, Bet v 6, Cup a 1, Pla a 1, Pla a 2, Pla a 3, Ole e 1, Ole e 7, Ole e 9, Phl p 1, Phl p 2, Phl p 5, Phl p 6, Phl p 7, Phl p 12, Lol p 1, Sal k 1, Pla l 1, Par j 2, Art v 1, and Art v 3. To evaluate mold sensitization, Alt a 1 and Alt a 6 were assessed [67]. Total IgE levels were reported in international units per milliliter (IU/mL), while sIgE levels were expressed in kU_A_/L, with values ≥ 0.3 kU_A_/L considered positive.

Considering the sIgE results, positive patients to any of the three most prevalent allergen sources in each study population were selected to prepare serum pools. The serum pools were prepared using an equal amount of these individual patient sera from each region (Barcelona, Granada, Lugo, Mostoles, Santiago, Sagunto, and Zaragoza). The optimal dilution of each serum pool was obtained by ELISA (approximately 1.5 of OD).

### 4.4. ELISA Assay

For the ELISA assay, Microlon high-binding plate wells (Greiner Bio-One, Frickenhausen, Germany) [68] were coated with 1 µg of allergenic extracts in sodium carbonate/bicarbonate buffer at 4 °C overnight (ON). After washing with phosphate-buffered saline (PBS), 0.25% Tween 20 (PBS-T) wells were blocked with PBS-T and 1% bovine serum albumin (BSA) for 3 h at room temperature (RT). After washing, 100 µL of different dilutions of serum pools in PBS-T-BSA were added and then incubated ON at RT. The bound IgE antibodies were detected by incubation with 100 µL of horseradish peroxidase (HRP)-conjugate anti human IgE (Southern Biotech, Birmingham, AL, USA) [69]. The substrate o-phenylenediamine dihydrochloride (Sigma-Aldrich, San Luis, MO, USA) [70] diluted in 10 mL of phosphate-citrate buffer 0.1 M, 0.025% peroxide hydrogen was added for the development of reactivity, and OD was measured in an ELISA reader at 405 nm (BioTek Synergy™ MX Multi-Mode Microplate Reader, Agilent Technologies, Santa Clara, CA, USA).

### 4.5. IgE Western Blot

Proteins from *D. pteronyssinus*, *L. destructor*, *Felis domesticus*, *Canis familiaris*, *Alternaria alternata*, *Cupressus arizonica*, *Olea europaea*, *Juniperus oxycedrus*, *Phleum pratense*, and *Quercus ilex* were separated by Any kD^TM^ Mini-PROTEAN^®^ TGX^TM^ precast polyacrylamide gels (Bio-Rad Laboratories, Hercules, CA, USA) [71] under reducing conditions according to the Laemmli’s method [72]. Proteins were electrotransferred to 0.45 µm nitrocellulose membranes, and the binding of IgE antibody to allergens was analyzed using pools serum of each region and anti-human IgE peroxidase conjugate (Southern Biotech, Biotechnology Research, Birmingham, AL, USA). Chemiluminescence detection reagents (Western Lightning PlusECL, Perkin Elmer, Walthman, MA, USA) were added following the manufacturer’s instructions, and the image was analyzed in Image Lab Touch software 3.0.1.14. IgE binding bands were identified using the BioRad Diversity database program (Bio-Rad Laboratories, Hercules, CA, USA) [71].

### 4.6. Statistical Analysis

Demographic characteristics were summarized using medians and standard deviations for continuous variables, and percentages for categorical variables. To compare differences, analysis of variance (ANOVA) was used for parametric continuous variables, the Kruskal–Wallis and Mann–Whitney U tests for nonparametric continuous variables, and the Chi-square test for categorical variables. A *p*-value of less than 0.05 was considered statistically significant. All statistical analyses were performed using GraphPad Prism version 10.0.0 for Windows (GraphPad Software, La Jolla, CA, USA) [73] and R version 4.4.2 for Windows (R Foundation for Statistical Computing, Vienna, Austria, Europe) [74].

## 5. Conclusions

In conclusion, this study provides a comprehensive overview of aeroallergen sensitization in pediatric populations across Spain. The regional variability in allergen exposure and age-related sensitization trends underscore the complexity of allergic diseases and the need for multifaceted management strategies. Clinicians should consider geographic factors when dealing with pediatric allergic diseases, as a deeper understanding of these patterns will enable more effective prevention, diagnosis, and treatment of allergic disorders, ultimately improving the quality of life for affected children and their families.

## Figures and Tables

**Table 1:** Descriptive statistics regarding basal comorbid conditions and associated clinical characteristics of the studied population.

	Barcelona, Cataluña	Granada, Andalucía	Lugo, Galicia	Móstoles, Madrid	Sagunto, Valencia	Santiago, Galicia	Valencia, Valencia	Zaragoza, Aragón
n = 236	27	30	30	29	30	30	30	30
Age (y.o.) median	9, 15	10, 23	10, 33	10, 34	9, 60	9, 33	10, 27	10, 60
<11 y.o. (n = 111)	18	15	15	15	17	19	15	15
≥11 y.o. (n = 125)	9	15	15	14	13	11	15	15
Sex (F/M)	9/18	16/14	12/18	11/18	10/20	9/21	11/19	10/20
Allergic Rhinitis (n = 205, %)	23 (85.2)	28 (93.3)	29 (96.7)	29 (100)	30 (100.0)	30 (100.0)	30 (100.0)	29 (96.7)
Allergic Asthma (n = 105, %)	15 (55.6)	23 (76.7)	15 (50.0)	15 (51.7)	17 (56.7)	10 (33.3)	9 (30.0)	16 (53.3)
SPT+ to any aeroallergen (%)	27 (100.0)	28 (93.3)	30 (100.0)	29 (100.0)	30 (100.0)	30 (100.0)	30 (100.0)	30 (100.0)
Total IgE (IU/mL) median	776.15	390.66	631.91	406.76	463.51	809.37	463.23	862.69

**Table 2:** Prevalence of sensitization to grouped local aeroallergens by the skin prick test (n = 236).

Positive SPT (n = 236)	Barcelona, Cataluña	Granada, Andalucía	Lugo, Galicia	Móstoles, Madrid	Sagunto, Valencia	Santiago, Galicia	Valencia, Valencia	Zaragoza, Aragón
HDM and/or SM (%)	26 (96.3)	2 (6.7)	28 (93.3)	7 (24.1)	20 (66.7)	19 (63.3)	22 (73.3)	10 (33.3)
Cat and/or dog dander (%)	9 (33.3)	14 (46.7)	9 (30.0)	16 (55.2)	15 (50.0)	9 (30.0)	8 (26.7)	24 (80.0)
Pollen (%)	15 (13.1)	28 (15.8)	16 (53.3)	28 (96.6)	23 (76.7)	23 (76.7)	16 (53.3)	30 (100.0)
Molds (%)	2 (7.4)	3 (2.2)	4 (13.3)	2 (6.9)	4 (13.3)	2 (6.7)	9 (30.0)	14 (46.7)

SPT: Skin Prick Test. HDM: House Dust Mites. SM: Storage Mites.

**Table 3:** Serological analysis—Mean (±standard deviation) of specific IgE levels (kU/L) to mite molecular allergens in patients from all eight centers. Values in parentheses indicate the number of sensitized patients (n) for each allergen.

Allergen	Barcelona	Granada	Lugo	Mostoles	Sagunto	Santiago	Valencia	Zaragoza
Der p 1	14.9 ± 18.7 (16)	0.9 ± 4.5 (2)	15.2 ± 20.0 (18)	0.4 ± 2.3 (1)	6.7 ± 12.6 (12)	11.0 ± 17.1 (14)	7.4 ± 13.6 (10)	4.5 ± 11.6 (5)
Der p 2	18.2 ± 18.7 (17)	2.6 ± 9.9 (2)	16.9 ± 21.6 (16)	1.6 ± 8.6 (1)	18.5 ± 21.0 (16)	18.1 ± 21.1 (18)	18.9 ± 22.3 (15)	5.6 ± 12.3 (8)
Der p 5	8.0 ± 14.3 (9)	0.0 ± 0.0 (0)	11.3 ± 19.2 (12)	0.4 ± 2.3 (1)	5.1 ± 11.6 (8)	9.3 ± 16.9 (11)	3.5 ± 9.4 (5)	0.0 ± 0.0 (0)
Der p 7	4.9 ± 11.5 (7)	0.0 ± 0.3 (1)	6.9 ± 15.4 (6)	0.2 ± 1.3 (1)	3.3 ± 8.2 (6)	7.2 ± 15.2 (7)	0.2 ± 0.9 (2)	0.4 ± 2.3 (1)
Der p 10	0.0 ± 0.0 (0)	0.0 ± 0.1 (1)	4.7 ± 12.8 (4)	0.0 ± 0.0 (0)	1.7 ± 9.1 (2)	0.3 ± 1.2 (2)	0.0 ± 0.0 (0)	0.0 ± 0.0 (0)
Der p 11	0.0 ± 0.0 (0)	0.0 ± 0.0 (0)	0.0 ± 0.0 (0)	0.0 ± 0.0 (0)	0.0 ± 0.0 (0)	0.0 ± 0.0 (0)	0.0 ± 0.0 (0)	0.0 ± 0.0 (0)
Der p 20	0.6 ± 2.2 (2)	0.0 ± 0.0 (0)	4.6 ± 11.8 (5)	0.0 ± 0.0 (0)	1.3 ± 5.7 (2)	0.0 ± 0.0 (0)	0.2 ± 1.1 (1)	0.0 ± 0.1 (1)
Der p 21	4.0 ± 10.8 (5)	0.0 ± 0.0 (0)	6.6 ± 13.1 (8)	0.0 ± 0.0 (0)	2.7 ± 9.6 (4)	5.4 ± 14.5 (6)	1.1 ± 4.0 (4)	1.6 ± 7.7 (2)
Der p 23	9.9 ± 11.6 (20)	0.3 ± 1.3 (2)	12.9 ± 17.8 (16)	0.0 ± 0.0 (0)	7.4 ± 12.7 (18)	9.2 ± 13.3 (20)	4.3 ± 7.7 (9)	2.1 ± 7.1 (3)
Der f 1	3.4 ± 8.2 (15)	0.1 ± 0.5 (1)	8.0 ± 14.0 (12)	0.4 ± 2.4 (1)	2.1 ± 3.4 (11)	5.7 ± 11.5 (10)	3.6 ± 7.4 (9)	0.5 ± 1.4 (5)
Der f 2	16.7 ± 18.5 (17)	2.0 ± 7.9 (2)	16.8 ± 21.3 (17)	1.3 ± 6.9 (2)	18.2 ± 21.5 (16)	14.2 ± 18.6 (18)	15.9 ± 19.7 (15)	5.8 ± 13.3 (7)
Blo t 5	1.4 ± 6.0 (3)	0.0 ± 0.0 (0)	0.2 ± 0.6 (2)	0.0 ± 0.0 (0)	1.0 ± 5.1 (2)	0.0 ± 0.1 (1)	0.0 ± 0.1 (1)	0.3 ± 1.7 (2)
Blo t 10	0.0 ± 0.0 (0)	0.0 ± 0.1 (1)	3.7 ± 10.3 (4)	0.0 ± 0.0 (0)	1.7 ± 9.1 (2)	0.2 ± 1.0 (1)	0.0 ± 0.0 (0)	0.0 ± 0.0 (0)
Blo t 21	0.1 ± 0.5 (1)	0.0 ± 0.0 (0)	0.1 ± 0.5 (2)	0.0 ± 0.0 (0)	0.3 ± 1.4 (1)	0.0 ± 0.0 (0)	0.0 ± 0.0 (0)	0.0 ± 0.0 (0)
Gly d 2	3.3 ± 10.0 (9)	0.0 ± 0.1 (1)	2.0 ± 6.2 (7)	0.2 ± 1.0 (1)	0.5 ± 1.3 (5)	0.7 ± 1.9 (6)	1.4 ± 5.0 (7)	0.2 ± 0.7 (2)
Lep d 2	1.8 ± 5.4 (7)	0.0 ± 0.2 (1)	4.2 ± 11.5 (9)	0.1 ± 0.6 (1)	0.1 ± 0.3 (2)	2.9 ± 9.0 (7)	0.3 ± 1.7 (1)	0.0 ± 0.1 (0)
Tyr p 2	1.8 ± 6.8 (4)	0.0 ± 0.0 (0)	3.2 ± 11.2 (5)	0.2 ± 0.6 (2)	0.1 ± 0.5 (1)	1.7 ± 7.3 (5)	1.1 ± 4.3 (2)	0.0 ± 0.1 (1)

**Table 4:** Serological analysis—Mean (± standard deviation) of specific IgE levels (kU/L) to pollen molecular allergens in patients from all eight centers. Values in parentheses indicate the number of sensitized patients (n) for each allergen.

Allergen	Barcelona	Granada	Lugo	Mostoles	Sagunto	Santiago	Valencia	Zaragoza
Bet v 1	0.0 ± 0.0 (0)	0.0 ± 0.2 (1)	4.1 ± 11.9 (5)	0.2 ± 0.7 (3)	0.3 ± 1.9 (1)	4.3 ± 13.6 (4)	0.0 ± 0.0 (0)	0.0 ± 0.0 (0)
Bet v 2	0.0 ± 0.0 (0)	0.7 ± 3.3 (5)	0.0 ± 0.0 (0)	0.1 ± 0.2 (2)	0.0 ± 0.0 (0)	0.2 ± 0.5 (4)	0.0 ± 0.1 (1)	1.9 ± 5.1 (6)
Bet v 6	0.0 ± 0.0 (0)	1.2 ± 2.7 (8)	0.0 ± 0.0 (0)	0.7 ± 3.5 (2)	0.0 ± 0.0 (0)	0.0 ± 0.0 (0)	0.0 ± 0.0 (0)	0.0 ± 0.0 (0)
Cup a 1	0.1 ± 0.2 (2)	3.2 ± 6.0 (17)	0.0 ± 0.0 (0)	3.6 ± 4.7 (16)	1.5 ± 4.0 (8)	0.0 ± 0.0 (0)	0.6 ± 2.2 (4)	6.5 ± 11.0 (22)
Pla a 1	0.0 ± 0.0 (0)	0.0 ± 0.0 (0)	0.0 ± 0.0 (0)	2.9 ± 10.0 (4)	0.0 ± 0.0 (0)	0.2 ± 1.1 (1)	0.0 ± 0.0 (0)	2.6 ± 7.8 (8)
Pla a 2	0.0 ± 0.0 (0)	0.7 ± 1.9 (8)	0.0 ± 0.0 (0)	1.8 ± 7.6 (7)	0.0 ± 0.0 (0)	0.0 ± 0.0 (0)	0.0 ± 0.0 (0)	0.8 ± 1.7 (9)
Pla a 3	0.3 ± 1.1 (2)	0.9 ± 4.4 (4)	0.0 ± 0.0 (0)	0.2 ± 0.5 (3)	0.2 ± 1.0 (3)	0.3 ± 1.0 (2)	0.2 ± 0.8 (2)	0.8 ± 2.1 (7)
Ole e 1	1.1 ± 3.7 (7)	24.4 ± 19.8 (26)	0.5 ± 2.5 (1)	23.4 ± 22.0 (20)	11.9 ± 18.8 (13)	1.1 ± 5.5 (1)	3.7 ± 9.2 (13)	13.2 ± 16.4 (18)
Ole e 7	0.0 ± 0.0 (0)	6.5 ± 12.3 (14)	0.1 ± 0.6 (2)	0.4 ± 1.5 (4)	0.0 ± 0.1 (1)	0.7 ± 2.3 (4)	0.0 ± 0.0 (0)	0.6 ± 1.4 (7)
Ole e 9	0.0 ± 0.1 (1)	8.8 ± 14.3 (10)	0.0 ± 0.0 (0)	1.9 ± 7.2 (3)	10.2 ± 17.3 (13)	0.0 ± 0.0 (0)	0.0 ± 0.1 (0)	0.1 ± 0.2 (1)
Phl p 1	2.5 ± 8.0 (4)	2.8 ± 7.9 (9)	11.7 ± 17.0 (18)	10.3 ± 15.3 (17)	4.4 ± 11.3 (7)	10.4 ± 18.1 (13)	1.4 ± 6.7 (2)	18.2 ± 18.8 (22)
Phl p 12	0.0 ± 0.0 (0)	1.5 ± 6.4 (5)	0.1 ± 0.3 (1)	0.2 ± 0.7 (3)	0.0 ± 0.0 (0)	0.6 ± 1.4 (4)	0.2 ± 0.8 (1)	3.3 ± 8.7 (6)
Phl p 2	0.1 ± 0.5 (1)	0.0 ± 0.0 (0)	6.0 ± 11.7 (11)	4.5 ± 11.2 (7)	0.5 ± 2.2 (3)	5.9 ± 12.7 (10)	0.0 ± 0.0 (0)	6.3 ± 14.7 (7)
Phl p 5	0.8 ± 2.9 (2)	1.6 ± 6.9 (4)	6.1 ± 13.6 (8)	8.2 ± 14.2 (12)	2.5 ± 9.7 (2)	10.5 ± 19.3 (8)	1.5 ± 7.6 (3)	11.0 ± 16.5 (15)
Phl p 6	1.0 ± 4.9 (2)	0.2 ± 0.8 (2)	2.5 ± 9.4 (7)	3.2 ± 8.6 (10)	0.1 ± 0.3 (2)	7.8 ± 16.7 (7)	0.2 ± 0.8 (1)	7.6 ± 14.2 (13)
Phl p 7	0.0 ± 0.0 (0)	1.3 ± 7.4 (1)	1.7 ± 9.1 (1)	3.6 ± 11.0 (3)	0.0 ± 0.1 (1)	3.3 ± 12.3 (2)	0.0 ± 0.0 (0)	0.4 ± 1.7 (2)
Lol p 1	2.2 ± 6.3 (4)	2.2 ± 6.5 (9)	6.4 ± 10.8 (16)	7.3 ± 12.3 (17)	1.3 ± 3.2 (7)	8.1 ± 14.8 (11)	0.7 ± 3.4 (2)	14.5 ± 14.2 (22)
Sal k 1	0.0 ± 0.0 (0)	7.0 ± 13.4 (13)	0.1 ± 0.4 (1)	1.7 ± 5.6 (5)	1.9 ± 6.1 (5)	0.0 ± 0.0 (0)	0.2 ± 0.9 (1)	13.0 ± 17.5 (16)
Par j 2	0.6 ± 3.2 (2)	0.1 ± 0.6 (1)	0.0 ± 0.0 (0)	0.0 ± 0.0 (0)	1.9 ± 7.7 (4)	0.3 ± 1.6 (1)	1.1 ± 4.8 (2)	0.0 ± 0.1 (1)
Pla l 1	0.0 ± 0.1 (1)	1.3 ± 6.7 (2)	0.0 ± 0.0 (0)	3.4 ± 10.0 (5)	0.0 ± 0.0 (0)	5.6 ± 13.8 (8)	0.0 ± 0.0 (0)	2.8 ± 7.6 (6)
Art v 1	0.0 ± 0.0 (0)	0.3 ± 1.7 (1)	0.0 ± 0.0 (0)	0.2 ± 1.1 (1)	0.0 ± 0.1 (1)	0.0 ± 0.0 (0)	0.0 ± 0.0 (0)	0.4 ± 1.7 (2)
Art v 3	0.1 ± 0.6 (1)	0.9 ± 2.5 (5)	0.0 ± 0.1 (1)	0.3 ± 1.1 (2)	0.1 ± 0.3 (1)	0.2 ± 0.9 (2)	0.2 ± 1.0 (2)	0.4 ± 1.2 (4)

## Data Availability

The data that support the findings of this study are available at each participating hospital, but restrictions apply to the availability of these data, which were used under license for the current study, and so are not publicly available. Data are, however, available from the authors upon reasonable request and with the permission of each hospital.
